# Supplementary material for: Rs11726196 Single-Nucleotide Polymorphism of the Transient Receptor Potential Canonical 3 (TRPC3) Gene Is Associated with Chronic Pain
Source: Int J Mol Sci. 2023 Jan 5;24(2):1028. doi: 10.3390/ijms24021028 (PMC9867099; doi:10.3390/ijms24021028)
Supplement: Supplementary file 1 [file ijms-24-01028-s001.zip › Supplementary files 221215/Supplementary Figure S1(GTEx Portal eQTL)_221006 (final).docx]

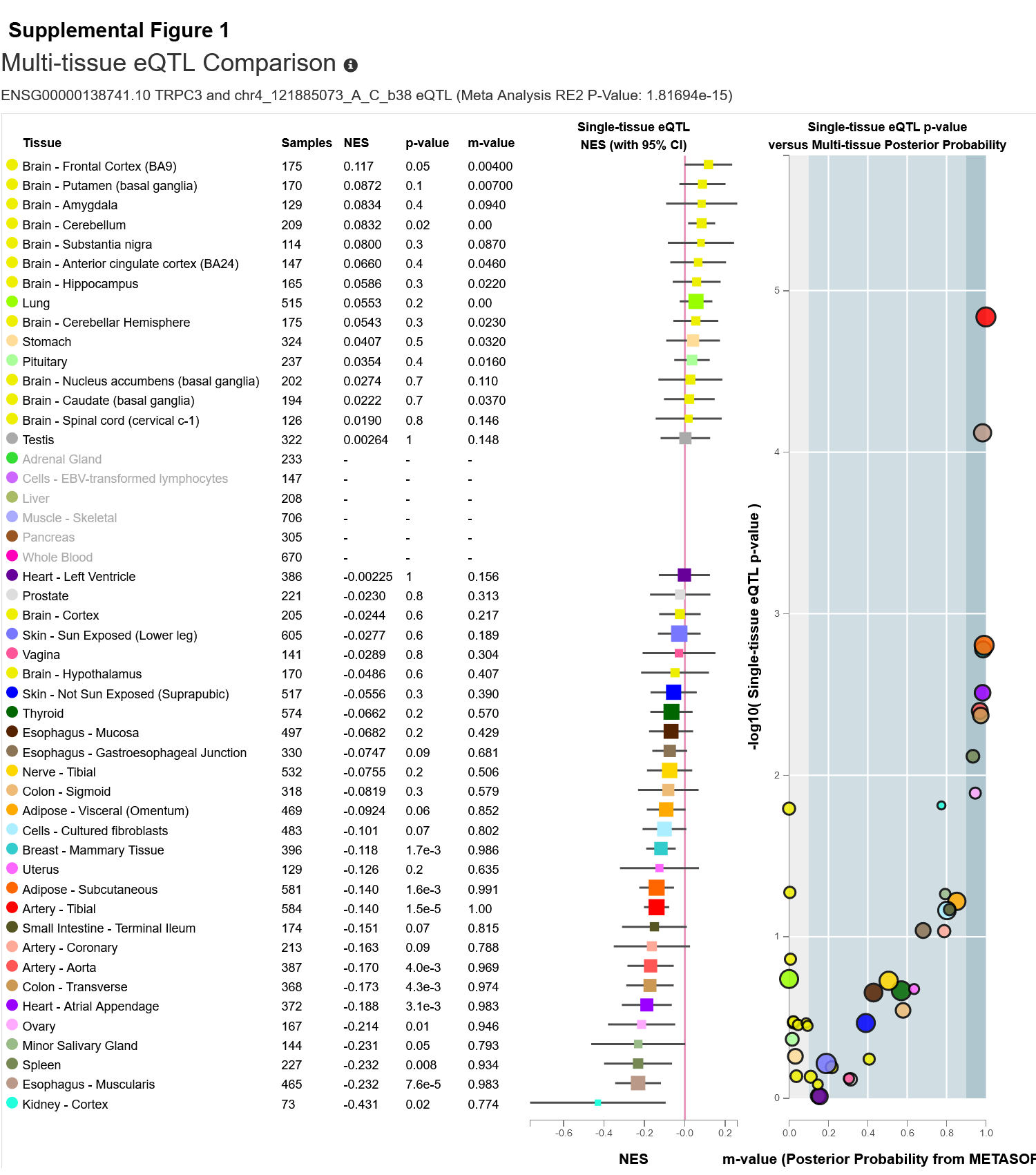


**Supplementary Figure S1. Multi-tissue eQTL comparison of *TRPC3* mRNA expression based on rs11726196 genotype.** Multi-tissue eQTLs of rs11726196, based on GTEx, are shown [23]. The normalized effect size (NES) of single-tissue eQTLs shows the genotypic trend of the amount of *TRPC3* mRNA expression in tissue. Positive NES values mean an mRNA expression trend of TT < TG < GG. Negative NES values mean an mRNA expression trend of TT > TG > GG.
